# Opioid prescribing practices prior to elective foot and ankle surgery: a population-based evaluation using health administrative data from a tertiary hospital in Canada

**DOI:** 10.1186/s12875-022-01722-x

**Published:** 2022-05-12

**Authors:** C. Michael Goplen, M. Elizabeth Pedersen, Ailar Ramadi, Lauren A. Beaupre

**Affiliations:** 1 Division of Orthopedic Surgery, Department of Surgery, Faculty of Medicine and Dentistry, University of Alberta, Edmonton, AB T6G 2R3 Canada; 2 Department of Physical Therapy, Faculty of Rehabilitation Medicine, University of Alberta, 6-110 Clinical Sciences Building, Edmonton, T6G 2G4 Canada

**Keywords:** Foot and ankle surgery, Opioid use, Pain management

## Abstract

**Background:**

Complex elective foot and ankle surgery is known to be painful so most patients are prescribed opioids at the time of surgery; however, the number of patients prescribed opioids while waiting for surgery in Canada is unknown. Our primary objective was to describe the pre and postoperative prescribing practices for patients in Alberta, Canada undergoing complex elective foot and ankle surgery. Secondarily, we evaluated postoperative opioid usage and hospital outcomes.

**Methods:**

In this population-based retrospective analysis, we identified all adult patients who underwent unilateral elective orthopedic foot and ankle surgery at a single tertiary hospital between May 1, 2015 and May 31, 2017. Patient and surgical data were extracted from a retrospective chart review and merged with prospectively collected, individual level drug dispensing administrative data to analyze opioid dispensing patterns, including dose, duration, and prescriber for six months before and after foot and ankle surgery.

**Results:**

Of the 100 patients, 45 had at least one opioid prescription dispensed within six months before surgery, and of these, 19 were long-term opioid users (> 90 days of continuous use). Most opioid users obtained opioid prescriptions from family physicians both before (78%) and after (65%) surgery. No preoperative non-users transitioned to long-term opioid use postoperatively, but 68.4% of the preoperative long-term opioid users remained long-term opioid users postoperatively. During the index hospitalization, preoperative long-term opioid users consumed higher doses of opioids (99.7 ± 120.5 mg/day) compared to opioid naive patients (28.5 ± 36.1 mg/day) (*p* < 0.001). Long-term opioid users stayed one day longer in hospital than opioid-naive patients (3.9 ± 2.8 days vs 2.7 ± 1.1 days; *p* = 0.01).

**Conclusions:**

A significant number of patients were dispensed opioids before and after foot and ankle surgery with the majority of prescriptions coming from primary care practitioners. Patients who were prescribed long-term opioids preoperatively were more likely to continue to use opioids at follow-up and required larger in-hospital opioid dosages and stayed longer in hospital. Further research and education for both patients and providers are needed to reduce the community-based prescribing of opioid medication pre-operatively and provide alternative pain management strategies prior to surgery to improve postoperative outcomes and reduce long-term postoperative opioid use.

## Introduction

Pain management among patients awaiting surgery for degenerative joint conditions, such as arthritis, is challenging [1]. These surgical candidates have typically exhausted nonsurgical treatments such as physical therapy and regular oral analgesia but still experience a stepwise deterioration in pain and function [2]. While Canada’s universal healthcare system provides equal access to elective surgery for all residents, system limitations such as access to operating room time can result in long wait times between referral and surgery [3]. This prolonged preoperative period can be challenging for primary care physicians who are left to manage patients’ pain associated with degenerative joint conditions such as arthritis [1, 4]. To bridge the gap between the time of referral and surgery, patients are often prescribed opioids to maintain function and improve pain, according to the World Health Organization Analgesic Ladder [5]. However, emerging evidence now suggests that opioids provide no benefit compared to other alternatives such as acetaminophen or ibuprofen [6, 7].

Patients prescribed preoperative opioids have more complicated hospital stays after surgery, increased rates of complications, and worse patient-reported outcome scores after surgery [8]. Further, communication among those providing hospital care and those providing care in the community is often limited or incomplete, leading to less than optimal patient care [9–11]. Currently, it is unknown if primary care physicians or subspecialists prescribe the majority of opioids before and after surgery. Without an understanding of prescribing practices, both before and after surgery, focused patient and physician education and implementation of standardized opioid prescribing practices for pre and postoperative pain management remains incomplete.

Thus, this study’s primary objective was to determine prescribing patterns six months (i.e., 180 days) before and after elective orthopedic foot and ankle surgery. It is also unknown if preoperative opioid use is associated with a) long-term opioid use that continues after recovery from the elective foot and ankle surgery is complete or b) increased opioid use in the hospital and increased health services use (e.g., length of stay, complications, re-admissions). Our secondary objectives were, therefore, to determine if long-term preoperative opioid use was associated with ongoing postoperative opioid use after index surgery relative to preoperative non-opioid users as well as the differences for in-hospital opioid requirements, hospital length of stay, complications, and readmission rates among preoperative opioid users and non-users.

## Methods

### Study design

This was a population-based retrospective study of all adults (> 18 years of age) who underwent elective foot and ankle surgery at a Canadian tertiary hospital. Four of the five (80%) fellowship-trained foot and ankle surgeons from this metropolitan health region operated out of this hospital, so although this was a single center evaluation, this hospital represented the majority of elective foot and ankle surgeries performed in the health zone during the evaluation time-period.

Potential participants were identified using ICD-10 procedural codes for ankle fusion (OSGF/OSGG), ankle replacement surgery (OSRF/OSRG) or hindfoot reconstruction (OSGH/OSGJ/OSSH/OSSJ) that were performed no later than 31May 2017. The selected end date allowed us to ensure all administrative data would be available to evaluate community-based opioid dispensing up to six-months postoperatively (i.e., Dec 2017). As there was a further delay in accessing administrative data of up to six-months to allow the data custodian to check data integrity, we performed our chart review in the summer/fall of 2018 to ensure a complete administrative dataset for opioid dispensing.

As we intended to review 100 patients’ charts using a retrospective hospital population-based approach, we identified that 100 patients received the surgical procedures of interest between May 1, 2015 and May 31, 2017 at our hospital. Although the sample size was not based on an apriori sample size calculation, there was no selection bias as we included all eligible surgeries within the evaluation period.

This study received ethics approval from the University of Alberta Health Research Ethics Board who provided waiver of written informed consent to allow access to routinely collected administrative health data (PRO00075984). All relevant guidelines regarding privacy and protection of health data were followed throughout the study.

### Phases of data collection

This study used 3 phases of data collection from 2 different data sources; the data sources are described below in detail. *Phase 1* was preoperative, which extended from 180 days preoperatively to the date of surgery. Data for this first phase came from our provincial community-based dispensing data system, the Pharmaceutical Information Network (PIN). As this was dispensing data, the preoperative opioid use data reflects estimates of consumption based on prescribing practices. As the PIN does not record hospital-administered medications, *Phase 2* of our data collection was the chart review where we were able to collect opioid consumption during the hospital stay as well as details around patients’ hospitalization. Finally, *Phase 3* repeated the data evaluation performed in Phase 1, but looked at PIN data for 180 days postoperatively, again allowing estimation of opioid use based on dispensing practices. Phase 3 started on Day 1 postoperative, but only included community-based dispensing of opioids (i.e., through the PIN) to understand postoperative prescribing practices after hospital discharge. No in-hospital consumption of opioids was included in the Phase 3 post-discharge evaluation, which continued for 180 days postoperatively.

### Data sources

#### Chart review

The chart review was conducted using a standardized data collection form completed by an experienced research associate. From the chart review, we were able to record the opioids consumed by patients during their hospital stay, which was transformed into a morphine equivalent dose (MED). Data on the participants’ age at surgery, sex, body mass index (BMI), smoking history, number and type of comorbidities, length of stay in hospital, destination of discharge (home versus rehabilitation hospital), postoperative complications, and readmission to hospital were also collected. For comorbidities, we specifically looked at recorded medical history to determine if any other chronic pain condition was identified and if the physician had identified that a patient was taking opioids for another condition.

#### Pharmaceutical Information Network (PIN)

Community-based opioid dispensing data were obtained from the PIN, a provincial pharmaceutical repository that maintains individual level pharmacotherapy records and includes dispensing information from all community pharmacies in Alberta, regardless of health insurance coverage.

A record was created in PIN each time a medication was dispensed from a pharmacy in Alberta and contained the drug information number (DIN), anatomic therapeutic code (ATC), date dispensed, dose and duration (days’ supplied) as well as prescriber classification. Each PIN entry was linked to patient’s Unique Lifetime Identifier (ULI), a unique number assigned to all persons who received health services in Alberta. Using opioid specific ATC codes, each patient’s PIN profile was queried for 180 days prior to and following the index surgery to determine their opioid dispensing history. The end-date for each dispensing was then calculated by adding the duration of the prescription to the dispensing date for each PIN entry as previously described [12].

Individual opioid prescriptions were then converted to a daily MED by multiplying the daily dose for each opioid by the corresponding MED to allow for standardized comparison across different opioid compounds [12]. Morphine conversion factors were based on the published conversion factors that align with Canadian opioid prescribing guidelines [7]. All datasets were deterministically linked using patient’s ULI that were previously scrambled with an algorithm that de-identified each ULI, but still preserved the ability to link across datasets.

### Classification of opioid use

*Preoperative long-term opioid users (OU)* were defined as patients who had 90-days or more of continuous opioid dispensing’s within 180 days prior to surgery. These parameters were consistent with the definition of long-term opioid therapy (LTOT) [12, 13]. *Preoperative intermittent OU* had recorded opioid dispensing’s within the 180 days prior to surgery, but did not meet the threshold parameters for a *preoperative long-term OU*. *Preoperative opioid naïve patients* did not have a recorded opioid dispensing within 180 days prior to surgery. The 180-day opioid free period had been previously used in studies investigating LTOT and is the established threshold for opioid discontinuation [14]. The maximum allowable refill gap between prescriptions in an opioid utilization episode was 14 days, or 0.5 times the preceding prescription length, whichever was greatest [12].

*Postoperative long-term OU*, *postoperative intermittent OU* and *postoperative non-OU* were defined using the same parameters as those used to define preoperative opioid use within 180 days after the index surgery, but did not include hospitalization opioid consumption.

### Statistical analysis

Descriptive statistics were used to characterize preoperative opioid dispensing patterns including dose (MED) and duration. Means with standard deviation (SD) or ranges were reported and were compared using Student’s t-test or one-way analysis of variance (ANOVA) for normally distributed variables. Ranges were selected to report PIN dispensing data while SD was used to report in-hospital opioid consumption. Categorical variables were represented as frequencies and proportions with Chi Square tests or Fishers Exact Test. Significance was set at *p* < 0.05 and statistics were performed using SAS (SAS institute), version 9.4.

## Results

Between May 1, 2015 and May 31, 2017, 100 patients underwent elective orthopedic foot and ankle surgery at the hospital of interest; 76 patients underwent ankle fusion, 21 received total ankle replacement and 3 underwent hindfoot reconstruction (Table 4—Hospitalization Data). The mean age of the study population was 61 ± 12 years, 46 were female and 17 had a history of smoking. The mean BMI was 32.5 ± 6.4 and 73 had two or more comorbidities. Patients prescribed long-term preoperative opioids had higher mean BMI (*p* = 0.05) and lower rates of smoking compared to intermittent users (*p* = 0.04), but no difference in number of comorbidities when compared to preoperative non-users and intermittent OU (Table 1).

**Table 1:** Pre-operative Characteristics

	**Preoperative Classification based on Community-Dispensing of Opioids via the Pharmaceutical Information Network (PIN)**			***P*-value**
	**Opioid Naive** **(*n* = 55)**	**Intermittent Opioid Users** **(*n* = 26)**	**Long-term Opioid Users** **(*n* = 19)**	
**Mean Age (SD)**	61.5 (12.9)	61.8 (13.1)	60.3 (10.1)	0.93
**Sex, n (%)**				
Female	25 (45.5)	11 (42.3)	10 (52.6)	0.77
Male	30 (54.5)	15 (57.7)	9 (47.4)	
**Mean BMI (SD)**	31.2 (5.5)	33.2 (6.7)	35.1 (7.4)	0.05
**Smoking history, n (%)**	5 (9.1)	8 (30.8)	4 (21.1)	0.04
**Comorbidities, n (%)**				
0–1	18 (32.7)	7 (26.9)	2 (10.5)	0.41
2–4	31 (56.4)	17 (65.4)	15 (78.9)	
> 4	6 (10.9)	2 (7.7)	2 (10.5)	

T-test or Wilcoxon ranks sum test was used for Age and BMI (continuous), and Chi-square test or Fisher’s exact test was used for categorical covariate as appropriate

*Legend:* *SD* Standard Deviation

Overall, 19 patients were considered long-term OU, 26 were intermittent OU and 55 were opioid naïve preoperatively. The majority of opioid users obtained their opioid prescriptions from family physicians both preoperatively (78%) and postoperatively (65%). Orthopedic surgeons did not prescribe to long-term OUs pre or postoperatively (Table 2).

**Table 2:** Community-Based Opioid Dispensing Before and After Elective Foot and Ankle Surgery

**Preoperative Community-Based Opioid Use**	**Preoperative Classification**			***P*-value**
	**Opioid Naive** **(*n* = 55)**	**Intermittent Opioid Users** **(*n* = 26)**	**Long-term Opioid Users** **(*n* = 19)**	
**Mean MED (range)**	0	10.9 (0.2, 110.3)	115.6 (3.3, 608.0)	< 0.001
**Mean Days of Opioid Use in past 180 days, (range)**	0	28.5 (4, 101)	147.3 (100, 183)	< 0.001
**Opioid Prescriber, n (%)**				
Family Physician	0	17 (65.4)	18 (94.7)	0.06
Orthopedic Surgeon	0	4 (15.3)	0 (0.0)	
Not Indicated	0	5 (19.2)	1 (5.3)	
**Postoperative Community-Based Opioid Use**	**Postoperative Classification**			***P*-value**
	**No Opioid Use Post Discharge** **(*n* = 13)**	**Intermittent Opioid Users (*n* = 72)**	**Long-term Opioid Users** **(*n* = 15)**	
**Mean MED (range)**	0	6.1 (0.75, 84)	153.5 (3.9, 448)	< 0.001
**Mean Days of Opioid Use 180 days post-surgery (range)**	0	13.7 (2, 90)	49.7 (30, 100)	< 0.001
**Opioid Prescriber, n (%)**				
Family Physician	0	44 (61.1)	13 (86.7)	0.065
Orthopedic Surgeon	0	20 (27.8)	0 (0.0)	
Not indicated	0	8 (11.1)	2 (13.3)	

T-test or Wilcoxon ranks sum test was used for Age and BMI (continuous), and Chi-square test or Fisher’s exact test was used for categorical covariate as appropriate

No preoperative naïve patients transitioned to long-term OU postoperatively, while 68.4% of the preoperative long-term OU remained long-term OU postoperatively. Of the preoperative intermittent OU, 88.5% continued to use opioids intermittently after hospital discharge (Table 3). Overall, the mean length of individual postoperative opioid prescription was 19 days (range 2 – 100 days) compared to 25 days (range 3 – 100 days) preoperatively, a 24% decrease in prescription duration.

**Table 3:** Change in Opioid User Status based on Community Dispensing of Opioids

Postoperative Classification	Preoperative Classification		
	**No Opioid Use** **(*n* = 55)**	**Intermittent Opioid Users** **(*n* = 26)**	**Long-term Opioid Users** **(*n* = 19)**
**No Post Discharge Opioid Use, n (%)**	12 (21.8)	1 (3.8)	0
**Intermittent Opioid Use, n (%)**	43 (78.2)	23 (88.5)	6 (31.6)
**Long-term opioid Use, n (%)**	0	2 (7.7)	13 (68.4)

During the hospital stay, preoperative long-term OU consumed higher doses of opioids (99.7 ± 120.5 mg/day) compared to both opioid naive (28.5 ± 36.1 mg/day) and intermittent OU (49.6 ± 69.0 mg/day). Further, long-term OU stayed on average one day longer in hospital compared to opioid naive patients (3.9 ± 2.8 days vs 2.7 ± 1.4 days; *p* = 0.012), were more likely discharged to a rehabilitation hospital (15.8% vs. 1.8%; *p* = 0.048) and had more postoperative readmissions (4 vs 2, *p* = 0.02) (Table 4).

**Table 4:** Hospitalization Data of 100 patients undergoing Elective Foot and Ankle Surgery

	**Preoperative Classification**			***P*-value**
	**Opioid Naive** **(*n* = 55)**	**Intermittent Opioid Users** **(*n* = 26)**	**Long-term Opioid Users** **(*n* = 19)**	
**Surgical Procedure, n (%)**				
Ankle Fusion	41 (74.6)	19 (73.1)	16 (84.3)	0.11
Total Ankle Replacement	13 (23.6)	7 (26.9)	1 (5.2)	
Hindfoot Reconstruction	1 (1.8)	0 (0)	2 (10.5)	
**Mean In hospital Opioid Consumption in MED (SD)**	28.5 (36.1)	49.6 (69.0)	99.7 (120.5)	< 0.001
**Mean length of stay in days (SD)**	2.7 (1.4)	2.5 (1.1)	3.9 (2.8)	0.01
**Discharge Location, n (%)**				
Home	54 (98.2)	25 (96.2)	16 (84.2)	0.048
Rehabilitation Hospital	1 (1.8)	1 (3.8)	3 (15.8)	
**Readmission to hospital, n (%)**	2 (3.6)	0 (0.0)	4 (21.1)	0.02
**Postoperative complications, n (%)**	14 (26)	5(19)	24 (24%)	0.80

## Discussion

A significant proportion of patients have opioids dispensed before surgery for degenerative joint conditions such as arthritis despite limited indications as outlined in the most recent Canadian Guideline for Opioids for Chronic Non-Cancer Pain [7, 8]. To our knowledge, this is the first report of prescriber patterns and the prevalence of opioid use before and after elective orthopedic foot and ankle surgery such as ankle fusions and replacement in Canada. Primary care providers were responsible for the majority of opioid prescriptions both pre and postoperatively. Preoperative opioid use among patients undergoing these foot and ankle surgeries was higher than other studies reporting rates of opioid use before total knee or hip replacement in Canada. Using the same parameters to define long-term OU, Goplen et al*.*(2021) reported that 31% of patients were dispensed opioids before hip or knee replacement surgery in Canada, and only 6.5% of these patients were considered long-term OU [15], compared to our reported rate of 45% and 19% respectively in this foot and ankle patient population. It is difficult to determine why opioid use differs so much between those undergoing foot and ankle surgery relative to joint replacement surgery. Published waiting times for foot and ankle surgery is not limited to elective procedures; further, these waiting lists only quantify time from the surgical consult to surgery and do not include the time that patients wait for the surgical consult, which can be substantial in Canada. However, when compared to hip and knee replacement waiting times (target < 6 months), patients in Alberta can wait for up to 46 weeks for foot and ankle surgery, which may have resulted in more opioids prescribed to patients for pain relief as they awaited surgery [16]. However, guidelines now emphasize limiting this practice [7]. It is now well established that opioids have no benefit than other alternatives such as acetaminophen or ibuprofen for such conditions as arthritis and have more complications [6, 7].

In our study, preoperative long-term OU’s stayed longer in hospital, were more likely to be discharged to a subacute hospital and had higher readmission rates; however, these results were not adjusted for group differences in BMI and smoking, so should be interpreted with a degree of caution. Unlike previously reported rates of opioid use for chronic non-cancer conditions [15], we were able to identify that the majority of opioids prescribed before surgery were by a primary care physician. These prescribing patterns follow professional guidelines that recommend their primary care provider prescribe opioids [17]. The results also highlight the family physician’s often overlooked role in optimizing patient outcomes as patients prescribed opioids have worse patient-reported outcomes after elective surgery [8, 18, 19]. Attempts to limit opioid exposure preoperatively could reduce the number of long-term postoperative users and improve post-surgical outcomes [20].

Our results were consistent with the reported finding that once patients have been prescribed opioids for more than 90 days, most continue to use opioids [13]. We found that most patients prescribed opioids before surgery continued using opioids at long-term follow-up; others have reported similar findings on ongoing postoperative opioid use despite reported improvements in pain and functional status [8]. This study also supports the previous reports that patients who are opioid naïve preoperatively rarely transition from short-term to long-term OU. No preoperative opioid naïve patients transitioned to long-term OU after surgery in our study.

Low-risk patients self-discontinue opioid therapy once acute postoperative pain subsides due to unwanted opioid side-effects or inadequate pain relief [21]. In contrast, patients considered high risk for persistent postoperative opioid use, such as those with a history of depression, anxiety, pain catastrophizing or substance use are thought to continue to use opioids due to complex interactions with the endogenous opioid system, which regulate both mood and pain perception [22–25]. Education regarding risk factors for LTOT based on preoperative opioid use could improve referral and triage practices for high-risk patients to minimize the number of patients initiated on preoperative opioid therapy before surgery [20, 23, 26, 27].

Finally, we observed that the mean duration of postoperative opioid prescription was reduced by 24% compared to preoperative prescriptions. This finding could be explained by standardized postoperative prescribing practices outlined in Canada’s opioid prescribing guidelines introduced during the study period [7]. These new guidelines recommended decreasing the duration and dose of opioid prescriptions to limit the potential for misuse and abuse [7].

Our study’s strength was our population-based approach to identify patients undergoing pre-specified complex elective foot and ankle surgeries and our ability to detect pre- and postoperative opioid dispensing using a provincial-wide pharmaceutical database and using accepted thresholds for long-term OU [12]. Our comprehensive database and our detailed methodology enabled detailed analysis of individual prescription’s formation, dose and duration as well as prescribers both preoperative and postoperatively for all opioids dispensed from community pharmacies in the Alberta.

This work was part of a multi-phase evaluation of current evidence and current practice patterns for pain management after elective foot and ankle surgery. Our initial scoping review found sparse evidence for pain management, with few randomized trials, all of which investigated short-term pain outcomes following a perioperative nerve block [28]. In addition, after this evaluation of local prescribing practices, we undertook an international Delphi Consensus process to develop consensus-based guidelines for pre-, peri- and post-operative pain management following elective foot and ankle surgery [29]. Using a multi-disciplinary group that included surgeons, pain physicians and anesthesiologists, community-based family physicians and allied health (physical therapists, psychologists, nurses), our guideline emphasized the need for opioid-sparing approaches, but also highlighted the urgent need for better communication between health care providers in the community and surgeons as well as patient-centric education regarding the use of opioids [29].

However, there are notable limitations of our evaluation including the assumption that opioid dispensing is a surrogate for patient consumption. It has been reported that administrative data is more accurate than patient-reported use; patients under-reported opioid use by as much as 46% prior to knee replacement due to the perceived stigma of disclosing opioid use to physicians [30, 31]. We were able to record the opioid consumption in hospital, but did not record opioid use during any rehabilitation stay. We also did not have access to the primary indication for opioid prescriptions. Although we collected comorbidity information specifically looking for other conditions that might be associated with opioid use or notation by the assessing physician that opioid use was for another condition, we were limited to information recorded in the chart. Thus, some patients may have had opioid prescriptions for unrelated chronic conditions. Further, as a secondary part of our evaluation, we did not risk-adjust the reported hospital outcomes, which may have been impacted by BMI or by type of procedure performed. Although no deaths were reported in the chart review, it is possible that deaths occurred post-discharge. Finally, this was also a small single centre study, but the selected hospital reflected the surgical practice of 4/5 foot and ankle surgeons in the health zone and the evaluation period captured all eligible foot and ankle surgeries that occurred.

## Conclusions

A significant number of patients continue to be dispensed opioids before and after complex elective orthopedic foot and ankle surgery by their primary care providers, despite limited indications. We observed that a substantial number of patients with preoperative opioid use continued to use opioids at six months after surgery. Further, they also used more opioids during their hospital stay and stayed longer in hospital.

These results provide valuable information to community clinicians who manage patients awaiting surgery and surgeons who counsel patients prior to surgery regarding expected outcomes and potential complications associated with preoperative opioid use. We hope that these results and our recently completed guidelines [29] will also facilitate improved communicate between surgeons and community physicians regarding managing these patients’ pain as they wait for surgery and then recover postoperatively. Further research on patient/provider education and pre-operative opioid-sparing management strategies are urgently needed to improve pre-operative management of these patients who experience prolonged wait times, while experiencing substantial pain and functional limitations.

## Data Availability

during the current study are not publicly available as our current ethics approval does not allow for data sharing but may available from the corresponding author on reasonable request with ethics amendment.

## Abbreviations

ANOVA: Analysis of Variance
ATC: Anatomic Therapeutic Code
BMI: Body Mass Index
95% CI: 95% Confidence Intervals
DIN: Drug Information Number
IQR: InterQuartile Rrange
LTOT: Long-term Opioid Therapy
MED: Morphine Equivalent Dose
OU: Opioid Users
PIN: Pharmaceutical Information Network
SD: Standard Deviation
ULI: Unique Lifetime Identifier

## References

[1] Boulanger A, Clark AJ, Squire P, Cui E, Horbay GLA (2007). Chronic pain in Canada: have we improved our management of chronic noncancer pain?. Pain Res Manag.

[2] Desmeules F, Dionne CE, Belzile E, Bourbonnais R, Frémont P (2010). The burden of wait for knee replacement surgery: effects on pain, function and health-related quality of life at the time of surgery. Rheumatology (Oxford).

[3] Canadian Institute for Health Information. Wait times for priority procedures in Canada. Ottawa: Canadian Institute for Health Information. 2017. https://secure.cihi.ca/free_products/wait-times-report-2017_en.pdf.

[4] Saïdi H, Pagé MG, Boulanger A, Ware MA, Choinière M (2018). Effectiveness of long-term opioid therapy among chronic non-cancer pain patients attending multidisciplinary pain treatment clinics: a Quebec pain registry study. Can J Pain.

[5] Cancer pain relief (1986). Geneva.

[6] Busse JW, Wang L, Kamaleldin M, Craigie S, Riva JJ, Montoya L (2018). Opioids for chronic noncancer pain. JAMA.

[7] Busse JW. The 2017 Canadian Guideline for Opioids for Chronic Non-Cancer Pain. Natl Pain Cent. 2017;1–105. Available from: http://nationalpaincentre.mcmaster.ca/documents/OpioidGLforCMAJ_01may2017.pdf.

[8] Goplen CM, Verbeek W, Kang SH, Jones CA, Voaklander DC, Churchill TA (2019). Preoperative opioid use is associated with worse patient outcomes after Total joint arthroplasty: a systematic review and meta-analysis. BMC Musculoskelet Disord.

[9] Rattray NA, Sico JJ, Cox LM, Russ AL, Matthias MS, Frankel RM (2017). Crossing the communication chasm: challenges and opportunities in transitions of care from the hospital to the primary care clinic. Jt Comm J Qual patient Saf.

[10] Vermeir P, Vandijck D, Degroote S, Peleman R, Verhaeghe R, Mortier E (2015). Communication in healthcare: a narrative review of the literature and practical recommendations. Int J Clin Pract.

[11] Solet DJ, Norvell JM, Rutan GH, Frankel RM (2005). Lost in translation: challenges and opportunities in physician-to-physician communication during patient handoffs. Acad Med.

[12] Goplen CM, Randall JR, Kang SH, Vakilian F, Jones CA, Voaklander DC (2019). The influence of allowable refill gaps on detecting long-term opioid therapy: an analysis of population-based administrative dispensing data among patients with knee arthritis awaiting total knee arthroplasty. J Manag care Spec Pharm.

[13] Von KM, Saunders K, Thomas Ray G, Boudreau D, Campbell C, Merrill J (2008). De facto long-term opioid therapy for noncancer pain. Clin J Pain.

[14] Vanderlip ER, Sullivan MD, Edlund MJ, Martin BC, Fortney J, Austen M (2014). National study of discontinuation of long-term opioid therapy among veterans. Pain.

[15] Goplen C, Kang S, Jason R, Donald V, Thomas C, Allyson J, et al. Preoperative long-term opioid therapy negatively impacts patient outcomes after total knee arthroplasty: an analysis of multicenter population-based administrative data. Can J Surg. 2021;64(2):E135–E143. 10.1503/cjs.007319 PMC8064248 33666382

[16] Alberta Wait Times Reporting (Online Source) Available at: http://waittimes.alberta.ca/ProcedureOverview.jsp. Accessed 20 Apr 2022.

[17] Physican C of P& S of A. Triplicate Prescribing Program Alberta. Available from: http://www.cpsa.ca/tpp/. Cited 2021 Jan 10.

[18] Ben-Ari A, Chansky H, Rozet I (2017). Preoperative opioid use is associated with early revision after total knee arthroplasty a study of male patients treated in the veterans affairs system. J Bone Jt Surg.

[19] Hansen CA, Inacio MCS, Pratt NL, Roughead EE, Graves SE (2017). Chronic use of opioids before and after total knee arthroplasty: a retrospective cohort study. J Arthroplasty.

[20] McAnally H (2017). Rationale for and approach to preoperative opioid weaning: a preoperative optimization protocol. Perioper Med.

[21] Noble M, Treadwell JR, Tregear SJ, Coates VH, Wiffen PJ, Akafomo C (2010). Long-term opioid management for chronic noncancer pain. Cochrane Database Syst Rev.

[22] Sullivan MD (2018). Depression effects on long-term prescription opioid use abuse, and addiction. Clin J Pain.

[23] Sullivan MD (2016). Why does depression promote long-term opioid use?. Pain.

[24] Sullivan MD, Ballantyne JC (2021). When physical and social pain coexist: insights into opioid therapy. Ann Fam Med.

[25] Sullivan MD, Edlund MJ, Zhang L, Unützer J, Wells KB (2006). Association between mental health disorders, problem drug use, and regular prescription opioid use. Arch Intern Med.

[26] Sullivan MD, Derbyshire SW (2015). Is there a purely biological core to pain experience?. Pain.

[27] Howe CQ, Sullivan MD (2014). The missing ‘P’ in pain management: how the current opioid epidemic highlights the need for psychiatric services in chronic pain care. Gen Hosp Psychiatry.

[28] Stiegelmar C, Li Y, Beaupre LA, Pedersen ME, Dillane D, Funabashi M (2019). Perioperative pain management and chronic postsurgical pain after elective foot and ankle surgery: a scoping review. Can J Anesth Can d’anesthésie.

[29] Dillane D, Ramadi A, Nathanail S, Dick BD, Bostick G, Chan K, Douglas C, Goplen G, Green J, Halliday S, Hellec B, Rashiq S, Scharfenberger A, Woolsey G, Beaupre LA PM. Elective Surgery in Ankle and Foot Disorders - Best Practices for Management of Pain: A Guideline for Clinicians. Can J Anesth Press. 2022. 10.1007/s12630-022-02267-4 35581524

[30] Stirratt MJ, Dunbar-Jacob J, Crane HM, Simoni JM, Czajkowski S, Hilliard ME (2015). Self-report measures of medication adherence behavior: recommendations on optimal use. Transl Behav Med.

[31] Hereford TE, Cryar KA, Edwards PK, Siegel ER, Barnes CL, Mears SC (2018). Patients with hip or knee arthritis underreport narcotic usage. J Arthroplasty.

